# Acute Popliteal Artery Occlusion after Revision Total Knee Arthroplasty

**DOI:** 10.1155/2015/672164

**Published:** 2015-08-19

**Authors:** Ryu Tsujimoto, Tomoyuki Matsumoto, Koji Takayama, Yohei Kawakami, Masato Kamimura, Takehiko Matsushita, Ryosuke Kuroda, Masahiro Kurosaka

**Affiliations:** Department of Orthopaedic Surgery, Kobe University Graduate School of Medicine, Kobe 28110, Japan

## Abstract

Acute arterial occlusions are a rare complication of total knee arthroplasty (TKA). However, in revision TKA, the risk of such complications is higher and these complications can lead to amputation if not adequately treated. We describe a case of acute popliteal artery occlusion 4 hours after second revision TKA in a patient with a history of several surgical procedures because of periprosthetic infection at a previous hospital. Revascularization was achieved via bypass grafting and amputation was narrowly avoided despite time lag after symptom onset to revascularization. In this case, it was possible that the arterial disease that accompanied the vascular endothelium injury such as pseudoaneurysm had existed since the previous surgery at another hospital and was destroyed by the surgical procedure, which led to the formation of thrombosis and arterial occlusion. Preoperative evaluation of the arterial condition should be considered to avoid acute arterial occlusive disease, especially in patients who had several previous surgical procedures.

## 1. Introduction

Primary total knee arthroplasty (TKA) is a very successful procedure that is becoming more widespread. However, surgeons are performing more revision TKAs because of patients' longer life spans. The reported 10-year survival rate in revision TKA was lower (86.1%–90.6%) compared with that of primary TKA (90.77%–98.1%) [1–5]. Furthermore, the clinical outcome of revision TKA is not as successful as that of primary TKA because the complexity of the revision procedure is associated with increased bone and soft tissue loss, which necessitates the use of larger and more constrained prostheses, along with other reasons [6].

Arterial complications after TKA are relatively rare, with a reported rate of 0.033%–0.17% in large patient populations [7, 8]. In an arthroplasty with complications such as revision, fracture, and infection, the risk of arterial events is increased [9]. Acute arterial occlusions are serious and can lead to amputation if inadequately treated. Treatment comprises anticoagulant use or surgical intervention, which includes thrombectomy and bypass grafting. Therefore, surgeons should take care to prevent the occurrence of this serious complication and avoid the worst scenario, amputation. Here, we report a case of acute popliteal artery occlusion after second revision TKA in a patient with a history of several surgical procedures because of periprosthetic infection, who avoided amputation but suffered ischemic lower limb paralysis.

## 2. Case Report

**Figure 1:**
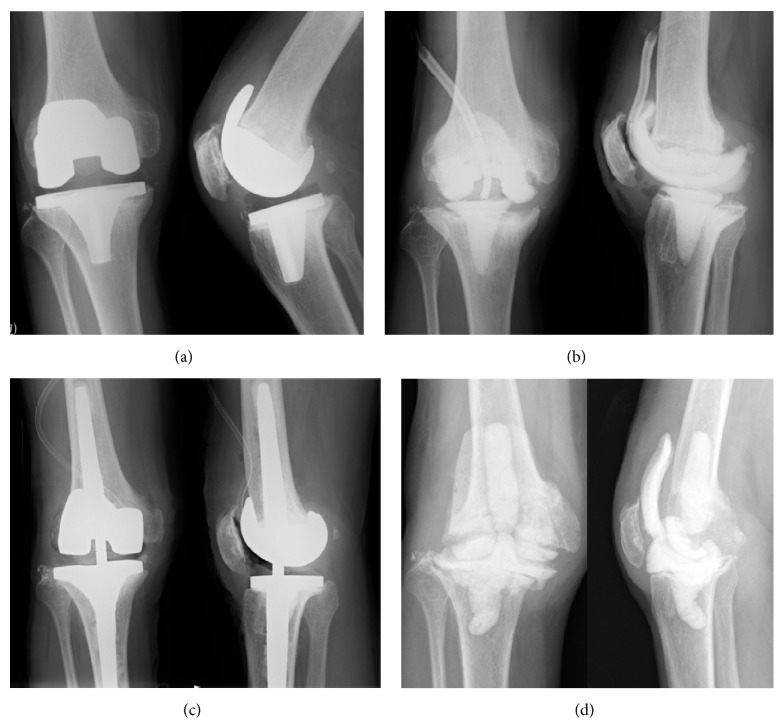
(a) Anteroposterior radiograph of right knee after primary TKA. (b) Anteroposterior radiograph of right knee after first antibiotic-impregnated cement spacer exchange and debridement. (c) Anteroposterior radiograph of right knee after revision TKA. (d) Anteroposterior radiograph of right knee after second antibiotic-impregnated cement spacer exchange and debridement.

An 83-year-old man presented to our university hospital because of uneradicated periprosthetic infection after TKA. He had undergone right primary TKA for incapacitating knee pain recalcitrant to conservative therapy at a previous hospital 35 months before visiting our hospital (Figure 1(a)). Eleven months after the first surgery, deep infection of TKA caused by methicillin-resistant* Staphylococcus aureus* (MRSA) occurred, so two-stage exchange arthroplasty (revision TKA following open irrigation and debridement of component retention) was performed 15 months after the first surgical procedure (Figures 1(b) and 1(c)), and treatment with vancomycin was employed. The patient received a blood transfusion of 1,680 mL red cell concentrates during the revision TKA. The infection recurred 1 month after revision TKA, and treatment was repeated with implant removal, debridement, and insertion of an antibiotic-impregnated cement spacer (Figure 1(d)). During this surgery, a blood transfusion of 2,800 mL was required again. However, the infection persisted, so the patient presented to our hospital 22 months after the primary TKA. Antibiotic-impregnated cement spacer exchange and debridement were repeated 7 months after revision TKA and eradication of infection was confirmed with aspiration in the outpatient clinic of our hospital more than three times. In addition, after completion of antibiotic therapy, the ESR, CRP level, and total WBC and differential counts in the joint aspirates were obtained, and the patient was observed for 2 more weeks. This was the fifth surgery the patient underwent.

The patient, 60 kg in weight and 156 cm in height, with a body mass index (BMI) of 25 kg/m^2^, had a history of hypertension, hypercholesteremia, and liver abscess and a 40-year smoking history. There was no history of ischemic heart disease or cerebrovascular problems, and there had been no symptoms of peripheral vascular dysfunction. He could walk with two canes. Preoperative knee motion with articulating cement spacer ranged from −5° of extension to 85° of flexion, and lag was 10°. Synovial fluid cultured by agar medium more than 7 days was negative, and blood C-reactive protein (CRP) level of and synovial leukocytes level was less than 1.0 mg/dL, 1000 cells/*μ*L, respectively, after the latest cement spacer prosthesis was implanted. All preoperative tests including liver function, renal function, complete blood count, and blood coagulation ability were within normal limits.

**Figure 2:**
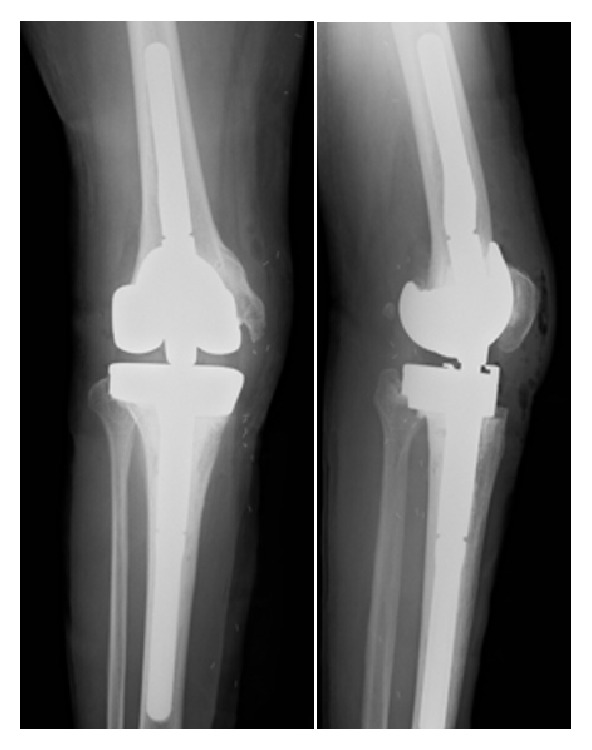
Postoperative anteroposterior radiograph of right knee.

**Figure 3:**
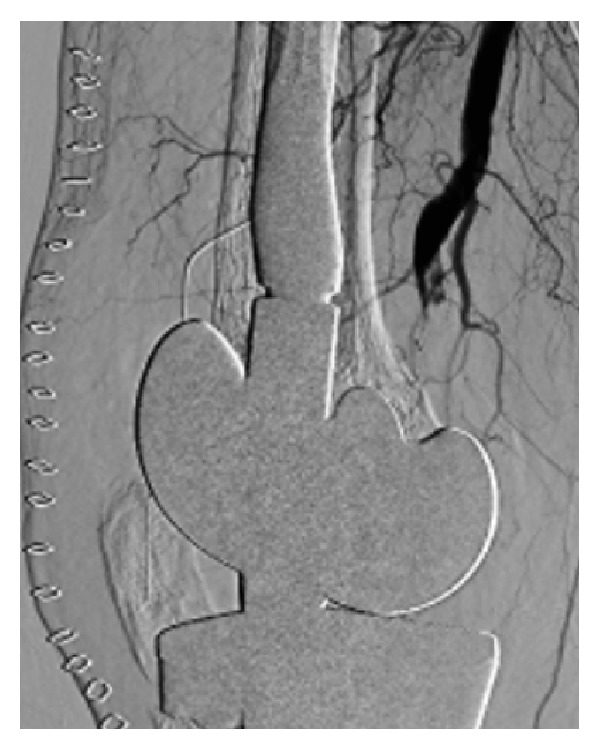
Arteriography of right knee showing the popliteal artery occlusion.

**Figure 4:**
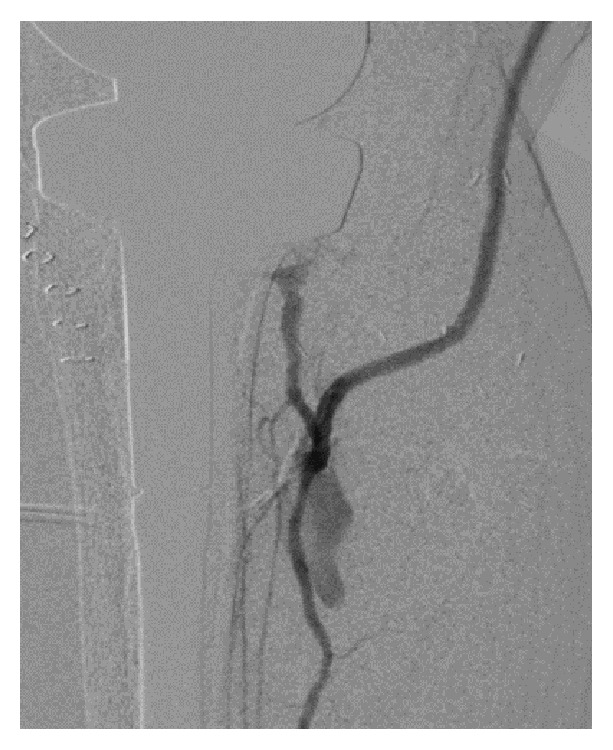
Arteriography of right knee (after bypass grafting) showing the recovery of lower limb circulation.

12 months after the last surgery, the second revision TKA was performed under general anesthesia, using a rotating-hinge knee implant (NexGen RH Knee, Zimmer, Warsaw, IN, USA). Prior to surgery, the limb was exsanguinated and the tourniquet was inflated to 280 mm Hg. The total tourniquet time was 167 minutes. The tourniquet was released before closing the surgical incision, and a 1200 mL hemorrhage was observed. The source of bleeding was unclear but stopped with hemostatic techniques and the surgery was completed (Figure 2). Immediately after surgery, the dorsalis pedis pulse weakened, and, four hours after surgery, the patient presented foot coldness, decreased sensation, and paresthesias; the dorsalis pedis pulse was not palpable. Emergency arteriography revealed arterial wall dissection and occlusion of the proximal popliteal artery (Figure 3). Thrombectomy with a Fogarty catheter was unsuccessful, so femoral artery to posterior tibial artery bypass with large saphenous vein graft and relaxing incision was immediately performed. The resumption of the lower limb circulation was confirmed by arteriogram 12 hours after the second revision TKA (Figure 4).

After surgery, wound healing was delayed and additional debridement and exchange of insert were undertaken 1 month after the rerevision TKA, and a full-thickness skin graft was performed 7 months after the second revision TKA. The patient was discharged from the hospital 12 months after the second revision TKA. Therefore, amputation was avoided, but drop foot by ischemic lower limb paralysis remained. However, he could walk with a cane and achieved active range of motion of the knee from −20° to 100°.

## 3. Discussion

According to a previous report, acute arterial occlusions after TKA are relatively rare. Rand reported 3 cases of acute arterial occlusions among 9,022 patients undergoing TKA (0.033%) [7], and Calligaro et al. reported 7 cases among 4,097 patients undergoing TKA (0.17%) [8]. In 31 cases of acute popliteal artery occlusion after TKA, 11 cases (35.5%) reportedly required amputation [10].

Patients of acute limb occlusion with severe symptom such as sensory loss of more than toe, rest pain, and moderate motor deficit require emergent surgical revascularization [11]. Emergency thrombectomy may be the initial step, and if thrombus removal is impossible, surgical bypass can be the likely choice [12]. In our case, the patient presented foot coldness, decreased sensation, and paresthesias, so the thrombectomy with a Fogarty catheter for revascularization of acute arterial occlusion was immediately attempted, and femoral artery to posterior tibial artery bypass with large saphenous vein graft and relaxing incision was performed because of failure of that trial.

The time from the end of surgery to the first symptom is important in revascularization of arterial occlusion. In a report by Green and Allen, 86% of the patients required amputation if the surgery was performed more than 8 hours after interruption of popliteal arterial blood flow [13]. In our case, surgery was performed 12 hours after revision TKA when the revascularization was completed by femoral artery to posterior tibial artery bypass. However, our patient narrowly avoided amputation. Emergency arteriography revealed that collateral circulation had already developed around the popliteal artery. We speculate that the peripheral blood flow was maintained because of this collateral circulation after the popliteal artery occlusion; therefore, the patient did not require amputation despite the time lag of revascularization.

It has been suggested that embolization resulting from disruption of atheromatous plaques causes acute popliteal artery occlusion. The risk factors of plaque disruption are peripheral vascular disease, tourniquet use in patients with calcification of the femoral or popliteal artery, and manual manipulation to reduce a flexion contracture [14–16]. Development of thrombosis caused by vascular endothelium injury and compression of the blood vessel by the surrounding soft tissue resulting from manual manipulation were also reported as causes of acute arterial occlusion [15, 17]. In our case, the patient had received a blood transfusion of 1,680 mL red cell concentrates during the first revision TKA and of 2,800 mL during the second implant removal and had undergone antibiotic-impregnated cement spacer placement before admission to our hospital. Additionally, the development of collateral circulation was seen during emergency arteriography. These facts indicate the possibility that arterial injury had been formed by surgical procedures of the previous surgeries, rub and push of cement spacer, and so on. Behnke et al. reported that, in the setting of a knee arthroplasty with infection, the risk of arterial events is increased [9], which supports our hypothesis. In the second revision TKA at our hospital, a 1200 mL hemorrhage was observed after tourniquet release; after the femoral-artery-to-posterior-tibial-artery bypass was completed, extravasation of contrast medium was observed in the distal part of the obstruction, which was speculated to be the injured part. Therefore, we suspect that arterial disease with vascular endothelial injury of popliteal artery such as pseudoaneurysm existed since the previous surgery. This part was destroyed by the operation in the second revision TKA, which led to the formation of thrombosis and arterial occlusion.

In conclusion, in the case of revision TKA in a patient with a history of several surgical procedures, arterial injury may have occurred. Therefore, the patient should be adequately evaluated for the existence of arterial disease before surgery, for example, by performing ultrasound and enhanced computed tomography. The preoperative evaluation of the patient's arterial condition should be considered to avoid acute arterial occlusive disease.
